# How to use prior knowledge and still give new data a chance?

**DOI:** 10.1002/pst.1862

**Published:** 2018-04-17

**Authors:** Kristina Weber, Rob Hemmings, Armin Koch

**Affiliations:** ^1^ Institute for Biostatistics Hannover Medical School Hanover Germany; ^2^ MHRA London UK

**Keywords:** Bayesian analysis, extrapolation, meta‐analysis, paediatric, prior knowledge

## Abstract

A common challenge for the development of drugs in rare diseases and special populations, eg, paediatrics, is the small numbers of patients that can be recruited into clinical trials. Extrapolation can be used to support development and licensing in paediatrics through the structured integration of available data in adults and prospectively generated data in paediatrics to derive conclusions that support licensing decisions in the target paediatric population. In this context, Bayesian analyses have been proposed to obtain formal proof of efficacy of a new drug or therapeutic principle by using additional information (data, opinion, or expectation), expressed through a prior distribution. However, little is said about the impact of the prior assumptions on the evaluation of outcome and prespecified strategies for decision‐making as required in the regulatory context.

On the basis of examples, we explore the use of data‐based Bayesian meta‐analytic–predictive methods and compare these approaches with common frequentist and Bayesian meta‐analysis models. Noninformative efficacy prior distributions usually do not change the conclusions irrespective of the chosen analysis method. However, if heterogeneity is considered, conclusions are highly dependent on the heterogeneity prior. When using informative efficacy priors based on previous study data in combination with heterogeneity priors, these may completely determine conclusions irrespective of the data generated in the target population. Thus, it is important to understand the impact of the prior assumptions and ensure that prospective trial data in the target population have an appropriate chance, to change prior belief to avoid trivial and potentially erroneous conclusions.

## 1 INTRODUCTION

In the planning of clinical trials, historical information based on other studies in the same treatment context is always indirectly incorporated in the study design, especially when choosing end points and justifying the assumptions for calculating the sample size. Bayesian analyses offer an opportunity to use existing information not only in the design but also in the analysis and hence in the decision‐making process since the Bayesian philosophy evaluates the outcome of a new experiment “in the light of” prior knowledge. This knowledge can be any information outside the current study that is formalized by means of parameters and distributions. It is often stated that incorporating historical (external) evidence increases the chance to provide formal proof of efficacy of a new drug or a therapeutic principle. This comes, however, at the price of introducing potentially undetected biases.^1^

While the Bayesian concept is generally applicable in the field of clinical trials, Bayesian methodology is currently discussed in situations with limited options to recruit patients into studies.^2^, ^3^, ^4^, ^5^ This is especially important in the context of rare disease. As, for example, in Hampson et al,^2^ the required specification of priors is often based on “expert opinion” to incorporate pathophysiological or pharmacological assumptions with the response parameter. This may be the only option in rare diseases to reduce the burden of evidence needed for proof of efficacy. Arguably, the motivation behind the use of Bayesian methodology is flawed, with historical information being incorporated in the search for a result that reaches the usual levels of evidence for regulatory decision‐making. It is more important that the strengths and weaknesses (uncertainties) in each individual piece of evidence are understood, even if usual standards cannot be met and decision‐making has to proceed under greater than usual uncertainty.

Another relevant area of application, on which the following chapters will mostly concentrate, is the study into “special populations,” for example, patients with decreased organ function, the elderly, and of greatest current interest the extrapolation of data generated in adults to paediatric patients. Extrapolation exercises can take different forms, although share a common goal to derive evidence in a target population that is fit for decision‐making without conducting stand‐alone studies in that target population to formally prove efficacy and safety. For an anti‐infective, where it can be confirmed that exposure‐response relationship will be the same regardless of the age of the patient, the evidence required to establish efficacy in children might be confirmation of a posology that gives rise to the desired exposure. In other cases, efficacy data generated in adults might be argued as being of some relevance to a target population (eg, adolescents), but additional prospective evidence should be generated in that target population, to make the totality of evidence compelling and/or to establish that effects in the target population are similar to those in the source population. A particular challenge for the use of quantitative approaches to synthesize data is that the amount of data proposed to be generated in the target population can be considerably lower than the amount already available in the source population. The European Medicines Agency has published a concept paper and 2 draft reflection papers on extrapolation of efficacy and safety in (paediatric) medicine development and proposes amongst others the use of adult information for the assessment of paediatric trials in a Bayesian approach.^6^, ^7^, ^8^ Two similar guidance documents were published by the Food and Drug Administration dealing with the application of the Bayesian concept to combine information from already available data and newly conducted studies in the field of paediatric studies and in medical devices clinical trials.^9^, ^10^

Three principles of drug regulation are challenged by the use of Bayesian methodology. Firstly, decision‐making should be based on data, which is interpreted in light of clinical or pharmacological plausibility and expert opinion. Secondly, an application is primarily constructed based on data for the drug itself; data from other drugs in the same class are of secondary interest. Finally, the decision for formal success or failure of each trial should be based on a prespecified primary analysis model and decision strategy so that conclusions cannot be influenced by post hoc choices. This is especially important if analyses are extended beyond Frequentist methodology into Bayesian approaches and informative priors are incorporated. It should be clear how the prior is chosen and the impact it has on the final conclusions.

Based on these principles, differences emerge between the use of Bayesian methods to support extrapolation and their use in rare diseases more generally. While instances exist where plausibility and expert opinion are the only options to justify the choice of priors for Bayesian analyses, for extrapolation adult data are available that are well understood and have justified licensing of the product. Here then, in contrast to de novo development in a rare disease, priors can be based on data that are justified as being of some direct relevance. While formally incorporating historical data into proof of efficacy involves assumptions and uncertainties, there is also an ethical mandate that available data should be used for decision‐making in the paediatric situation to avoid unnecessary experimentation in a vulnerable population. The question then turns to the methodologies available to combine historical and prospective data in a structured manner.

Pocock^11^ discussed prerequisites to be fulfilled if one study should be used to derive prior information for another study: Both studies should investigate the same control and active treatment and should be of similar design and conduct, meaning that the study protocol, the clinical investigators, and the principal company should be the same. In a best‐case scenario, the inclusion and exclusion criteria in both studies will be alike so that the trial therapies are investigated precisely in the same population. Intuitively, these criteria seem reasonable, but they cannot be fulfilled in the field of paediatric extrapolation. This clarifies that more assumptions have to be met once adult data are used to construct a prior for use in clinical trials in paediatrics. Indeed, in addition to having discussed the potential for differences in disease, drug pharmacology, and/or clinical response between adults and children, it is also necessary to consider sources of heterogeneity between the historical and prospective trial data, eg, the impact of the treating physician and trial conduct.

In line with the principles described above, our interest is to explore the use of data‐based priors in the decision‐making process of paediatric extrapolation. For the sake of completeness, we compare this approach with direct frequentist and Bayesian meta‐analytical approaches that could also be used to combine available adult and newly generated paediatric data to arrive at a summary conclusion.

The outline of the paper is as follows: In Section 2, we start with a classical example from meta‐analysis in drug licensing where 2 similar sized trials addressing the same question showed contradictory findings. This example is used to investigate Bayesian analyses in a scenario of having equal amounts of information in the prior and in the prospective data. This is taken as a start to further investigate the impact of unequal trial sizes as is usually the case in the extrapolation context. In the second example, representing the typical extrapolation framework, 3 successful studies in adults are available, and one study in paediatrics is planned. This will be used to experiment with paediatric study results that are (1) in line with the adult study outcome and (2) relevantly different from the adult information. Section 3 gives an overview of the applied methods and analyses. We compare the results in different Bayesian and frequentist approaches. We conclude with a discussion about key elements for the planning of such extrapolation exercises in an attempt to guarantee that prior knowledge is incorporated in a way that avoids trivial conclusions completely determined by the already available adult data. Whenever decision‐making strategies are discussed, credible intervals (in the Bayesian framework) and confidence intervals (in the frequentist framework) will be used interchangeably.

## 2 MOTIVATING EXAMPLES

### 2.1 Bond and Opera in functional dyspepsia

Bond and Opera are 2 trials comparing omeprazole with placebo in functional dyspepsia.^12^ Both studies are double‐blind, randomized, placebo‐controlled of almost equal size and randomized to either treatment with omeprazole 20 mg, omeprazole 10 mg, or placebo. The main outcome of the studies is the proportion of patients with complete symptom relief in each of the treatment groups. In the following, we concentrate on the comparison of the high‐dose omeprazole and the placebo group. In line with the Pocock criteria, both studies investigated the same treatment in the same indication and were planned by the same company with almost identical study protocols apart from the conduct in different regions of the world. Event numbers, sample sizes, and individual trial treatment effects, measured on the log odds ratio (OR) scale, are presented in Table 1. In the first study, Bond, a large treatment effect with a treatment difference of 16.5% or a log OR of 0.74 was observed in the omeprazole 20 mg group. This effect is significant at the nominal level of 5% (*P* < .001) and, therefore, can be seen as evidence of efficacy in the population under investigation. In contrast, almost no effect of omeprazole was observed in the Opera study: Only 33.7% of the patients treated with omeprazole reached the end point compared with 30.5% on placebo (*P* = .25). Although both studies were designed and executed similarly, different conclusions about evidence of efficacy have to be drawn depending on the study results. This circumstance questions all attempts to combine these studies.

**Table 1:** Overview of event data and sample size in the Bond and Opera studies

Study	Omeprazole 20 mg Events/Treated	Placebo Events/Treated	Log OR	95% CI	*P* Value
Bond	93/219 (42.5%)	57/219 (26.0%)	0.74	(0.32 to 1.15),	<.001
Opera	68/202 (33.7%)	62/203 (30.5%)	0.14	(−0.27 to 0.55)	.252

### 2.2 Paediatric extrapolation for immunosuppressive treatment after kidney transplantation

In the previous example, an equal amount of information was available from both studies. In extrapolation between adults and paediatrics, it is usually the case that a substantial amount of information from one or more adult studies is available. This greater amount of information, if weighted equally, would dwarf the limited information from a single trial in paediatrics. Studies of immunosuppressive treatment in kidney‐transplanted patients are considered as an example. To compare with the first example, we display results here in the same metric.

Three adult studies compared the experimental compound with a standard of care regimen in adult patients.^13^, ^14^, ^15^ The objective of all studies is to show noninferiority of a treatment with the new immunosuppressive compound (usually as part of a combination treatment) to a standard of care combination treatment. A composite primary end point reflecting treatment failure was used in all studies and included biopsy‐proven acute rejection, graft loss, death, or loss to follow‐up. Table 2 summarizes the studies and provides the overall treatment effect in the adult population with results of fixed‐effect and random‐effects meta‐analyses. All adult studies were planned with similar sample sizes to demonstrate noninferiority to control with a noninferiority margin of 10%. Based on the observed control effect, this noninferiority margin can be translated into roughly 1.54 on the OR scale or 0.43 on the log OR scale. A justification of this noninferiority margin was not directly provided in the publications, and we assume it to be based on the rarity of the condition under investigation rather than usual statistical and clinical considerations, although such an approach cannot be supported, in general. Due to the low heterogeneity, the fixed‐effect and random‐effects meta‐analysis estimate the same overall effect. The overall effect is close to zero, and therefore, noninferiority and even equivalence between the new and the control strategy can be demonstrated in adult patients.

**Table 2:** Adult studies in the immunosuppressive treatment example

Study	EVR Events/Treated	MPA Events/Treated	Log OR	95% CI	*P* Value
Vitko et al^13^	58/194 (29.9%)	61/196 (31.1%)	−0.05	(−0.48 to 0.38)	.793
Lorber et al^14^	48/193 (24.9%)	54/196 (27.6%)	−0.13	(−0.58 to 0.32)	.548
Tedesco Silva et al^15^	70/277 (25.3%)	67/277 (24.2%)	0.06	(−0.33 to 0.45)	.844
Meta‐analysis FEM & REM			−0.035	(−0.28 to 0.21)	.776

A paediatric investigation plan has been initially agreed on a randomized clinical trial including 53 paediatric patients per treatment group and comparing a regimen including the experimental compound with a standard paediatric regimen, for which agreement with experts has been achieved. In the following, we critically discuss the behaviour of various approaches to demonstrate efficacy in the paediatric population based on an extrapolation approach.

In this experiment, we evaluate the results of the adult studies together with the fictional scenarios about the paediatric trial as outlined in Table 3. The first scenario assumes homogeneity of the treatment effect in adult and paediatric studies. The second scenario is chosen to represent heterogeneity with a true log OR marginally greater than the noninferiority margin implying that the hypothesis of inferiority of the experimental drug should not be rejected based on the paediatric trial.

**Table 3:** Possible outcomes of the paediatric study

Study	EVR Events/Treated	MPA Events/Treated	Log OR	95% CI	*P* Value
Scenario 1	16/53 (30.2%)	16/53 (30.2%)	0.00	(−0.83 to 0.83)	1.00
Scenario 2	22/53 (41.5%)	16/53 (30.2%)	0.50	(−0.31 to 1.30)	.33

## 3 METHODS

### 3.1 Statistical data model

We consider the situation where a new treatment *T* is compared with a control treatment *C*, either standard of care or placebo. The outcome in both treatment arms is a binary variable and binomially distributed with event rates *pT* and *pC* and sample sizes *nT* and *nC*.The treatment effect in the control group can be derived based on the event rate logit in the control group

$$\mu =\text{logit}\;\left(\mathrm{pC}\right).$$

The effect in the treatment group *δ* is additive on the logit scale and estimated using a log OR

$$\text{logit}\left(\mathrm{pT}\right)=\mu +\delta .$$

Parameters of a similar deemed historical trial will be represented using a subscript *H*. If all studies are included in a meta‐analysis at once, the subscript *i* = 1, …, *n* is used as a study identifier.

### 3.2 Meta‐analysis

There are several ways to summarize the information of at least 2 trials and arrive at a summary conclusion on the treatment effect. The most common approach is a meta‐analysis that is often conducted in a fixed‐effect or random‐effects model.

It is possible to implement both of these meta‐analysis models in a frequentist and a Bayesian framework. In the Bayesian framework, it is necessary to specify prior distributions for all unknown parameters. These priors lead in combination with the data to a joint posterior density function that can be used to derive conclusions about the parameter of interest by obtaining the marginal posterior densities. Prior distributions used in this work are described in Section 3.4. Whitehead^16^ showed that the posterior distribution cannot be easily derived and the integrals defining the marginal posterior distributions cannot be calculated in closed form. Thus, we used Gibbs sampling and the DIRECT algorithm^17^ to derive posterior distributions for all unknown parameters in the Bayesian framework using R^18^ and the bayesmeta^19^ and R2WinBUGS^20^ packages.

#### 3.2.1 Frequentist and Bayesian fixed‐effect models

A fixed‐effects meta‐analysis assumes no heterogeneity between studies, and the estimated study‐specific treatment effects ${\hat{\delta }}_{i}$ are normally distributed around a common treatment effect *δ* by

$${\hat{\delta }}_{i}\sim N\left(\delta ,\sigma_{i}^{2}\right).$$

The within‐study variance $\sigma_{i}^{2}$ is treated as known, using the variance of the observed effect. The common treatment effect in the frequentist fixed‐effect meta‐analysis is estimated by an inverse variance–weighted method with weights $w_{i}=1/\sigma_{i}^{2}$.^16^ The pooled treatment effect of *n* contributing studies is estimated by $\hat{\delta }=\sum_{i=1}^{n}w_{i}{\hat{\delta }}_{i}/\sum_{i=1}^{n}w_{i}$ with variance $1/\sum_{i=1}^{n}w_{i}$. In the Bayesian framework, the fixed‐effect model leads to the same distributional assumption on the treatment effect posterior as the frequentist framework, if uninformative priors on the unknown parameters are implemented and the variance is assumed to be known.

#### 3.2.2 Frequentist and Bayesian random‐effects meta‐analyses

In the random‐effects model, within‐study variability $\sigma_{i}^{2}$ and between‐study variability *τ*^2^ (heterogeneity) is assumed, and the distribution of the observed study‐specific effects is

$${\hat{\delta }}_{i}\sim N\left(\delta ,\sigma_{i}^{2}+\tau^{2}\right).$$

The overall effect ${\hat{\delta }}^{*}$ and its variance can be estimated as in the fixed‐effect model with adjusted weights $w_{i}^{*}=1/\left[\sigma_{i}^{2}+\tau^{2}\right]$. Thus, the distributional assumptions of the random‐effects and the fixed‐effect model are equal if the heterogeneity parameter equals zero. In practice, estimation of the heterogeneity parameter will lead to different results. It is often said that if a positive estimate for the between‐study variance is calculated, the estimates of both approaches will differ leading to a more conservative confidence interval for the common effect estimate in the random‐effects model.^21^

In the Bayesian random‐effects meta‐analysis, all model parameters and the data are treated as random, and the distributional assumption of the true study‐specific effects is

$$\delta_{i}\sim N\left({\hat{\delta }}^{*},1/\sum_{i=1}^{n}w_{i}^{*}\right).$$

If *τ*^2^ is known, there is no difference between the frequentist and Bayesian distributional assumption. Since this is normally not the case, the heterogeneity “is simply treated as another unknown parameter to be estimated, and the posterior of the parameter of interest will automatically incorporate the uncertainty in between‐study variance.”^22^

### 3.3 Bayesian meta‐analytic predictive approach

In a Bayesian meta‐analytic predictive (MAP) analysis, 2 steps are necessary. First, the posterior‐predictive distribution of a meta‐analysis treatment effect has to be derived and can be used, secondly, as a prior distribution on the treatment effect in a new study.^23^ This method uses one of the key features of the Bayesian framework: “the incorporation of subjective, or data‐based, prior beliefs into the analysis of data.”^24^ This seems aligned to the concept of extrapolation whereby, if available information on differences in disease, drug pharmacology, and/or clinical response permits, results in the source population can be used to make predictions about likely effects in a target population. The incorporation of historical control data in a MAP approach has already been proposed.^4^ It should be noted that this is a different way of summarizing available treatment information in comparison with meta‐analyses. In the meta‐analysis framework, the goal is to estimate a common unknown treatment effect from several studies. The MAP approach estimates the unknown effect of one study “in light of” another study or more specifically update the prior belief in light of new evidence. This approach is possible using both the fixed‐effects or random‐effects assumptions.

#### 3.3.1 Fixed‐effect MAP approach

In case only one study is available and the rather strict fixed‐effect assumption holds true, the posterior‐predictive distribution of the treatment that is used as the prior distribution of the treatment effect in a new study can be specified as

$$\delta \left|\delta_{H}\sim N\left(\delta_{H},\sigma_{H}^{2}\right).\right.$$

If more than one study is available, it is possible to use a meta‐analysis estimate of the overall effect and the corresponding variance in the equation above. The prior and the new study data are thereafter used to form a posterior distribution of the treatment effect in the paediatric trial.

#### 3.3.2 Random‐effects MAP approach

In the random‐effects model, both study effects are exchangeable and random variables of the same normal distribution

$$\delta ,\delta_{H}\sim N\left(\theta ,\tau^{2}\right).$$

If *τ* is known, the predictive distribution of a study treatment effect in a new trial given the historical trial is of the form^23^

$$\delta \left|\delta_{H}\sim N\left(\delta_{H},2\tau^{2}+\sigma_{H}^{2}\right)\right..$$

Since in most cases *τ*^2^ is not known, a prior distribution for this parameter has to be specified in the analysis of the historical trial to obtain the posterior‐predictive distribution of *δ*. In case of several historical trials, the predictive distribution hales from a random‐effects meta‐analysis of all historical trials. The predictive distribution incorporates the heterogeneity between the observed and all future trials. In the second step, this predictive distribution is used as a treatment effect prior in the analysis of a new study. In this step, a heterogeneity prior is no longer needed for the derivation of the posterior based on the predictive prior distribution and the new data. This model and the subsequent use of the historical trial are described as “dynamic borrowing” in case of incorporation of already existing control data.^1^ If the fit of the historical data to the current data is good and the data are consistent, then the heterogeneity parameter should be small and will lead to a higher borrowing of the historical data in the current analysis. On the other hand, if something in the data speaks against consistency, then the heterogeneity parameter should get bigger and the historical data are downweighted. This reduces the amount of borrowing and impacts the power of the prospective trial and the resulting integrated analysis.

The power prior approach^25^ is a similar approach that leads to a downweighting of already existent data by a chosen proportion. It has already been shown that there is no real difference between a random‐effects meta‐analytic approach and the use of a power prior.^26^, ^27^ The main difference is the discounting of prior information that is based on an arbitrarily chosen factor.

### 3.4 Choice of prior distributions for the heterogeneity parameter

Based on the statistical data model in Section 3.1, it is necessary to choose a prior distribution on the treatment effect and the study‐specific control effects in all models. To make sure that the choice of the distribution does not impact on the estimate of the treatment effect, it is important to choose uninformative or at least vague prior distributions. Normally distributed uninformative or vague priors for these effects are defined as^28^

$$\mu_{i}\sim N\left(0,\;4\right)\;\text{and}\;\delta \sim N\left(0,\;10\right).$$

In the random‐effects model, we investigate the influence of 5 different heterogeneity priors based on the 2‐parameter inverse‐gamma (IG) and the 1‐parameter half‐normal^5^ distribution representing different beliefs of heterogeneity between the studies. Table 4 gives an overview of the assumptions on heterogeneity by translating the chosen priors into the expected ratio between the ORs of 2 independent trials in a meta‐analysis with the prior mean as observed heterogeneity. The range of possible ratios lies between 1, corresponding to no heterogeneity at all, and 23, corresponding to substantial heterogeneity. These prior distributions are all informative and incorporate additional information not obtained from the trials that are used in the Bayesian analyses. It is important to use informative prior distributions for the heterogeneity parameter where only very limited information on between‐study heterogeneity is provided by the studies themselves. Additionally, Higgins and Whitehead^29^ showed that a noninformative IG prior on the heterogeneity parameter does not lead to a satisfactory convergence of the Markov chain, which we also observed in our analyses when implementing an IG prior with equally small scale and shape parameters (not shown here). Moreover, Lambert et al^30^ investigated in a simulation study several alleged noninformative priors on the heterogeneity parameter in a random‐effects model. They concluded that, especially in small meta‐analyses, conclusions from the same data can differ depending on the chosen prior specification, although all priors were assumed to be noninformative.

**Table 4:** Prior assumptions on the distribution of the heterogeneity parameter

Heterogeneity Prior	E(***τ***^**2**^)	*OR*_**1**_/***OR***_**2**_
on *τ*^2^		
IG(1/3,1)	0.33	10
IG(1/7,1)	0.14	4
IG(1/1000,1)	0.001	1
on *τ*		
HN(1)	0.61	23
HN(0.5)	0,16	5

## 4 RESULTS

**Figure 1:**
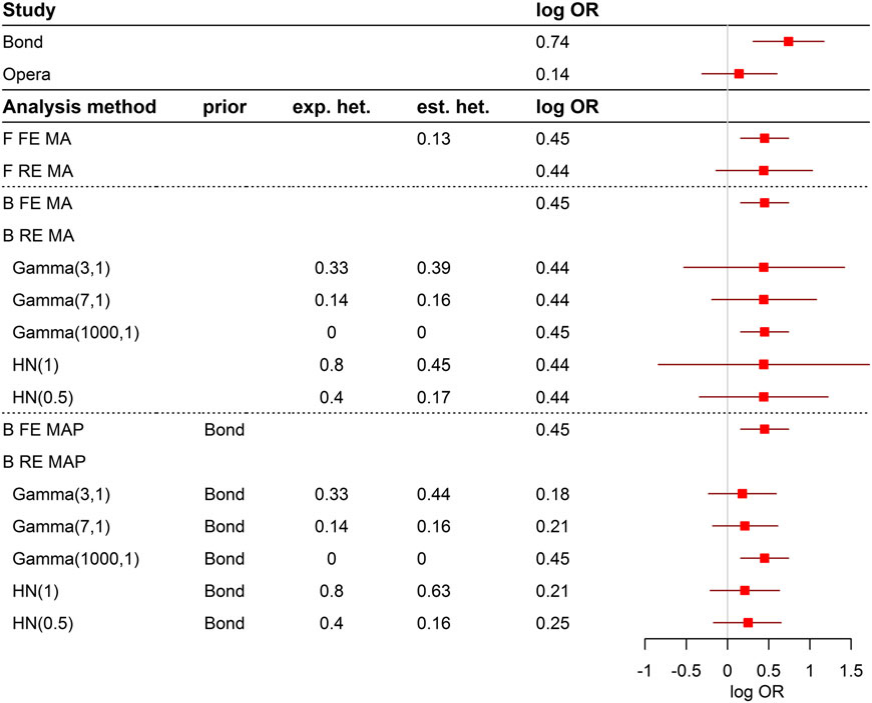
Results and conclusions for the Bond and Opera studies

**Figure 2:**
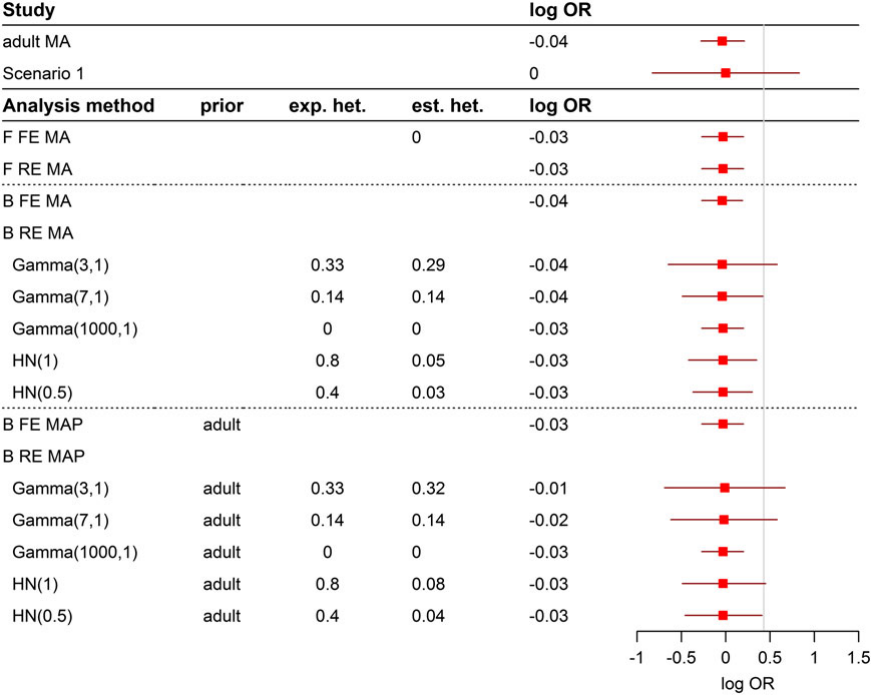
Results and conclusions for the comparison of immunosuppressive strategies in the homogeneous case

**Figure 3:**
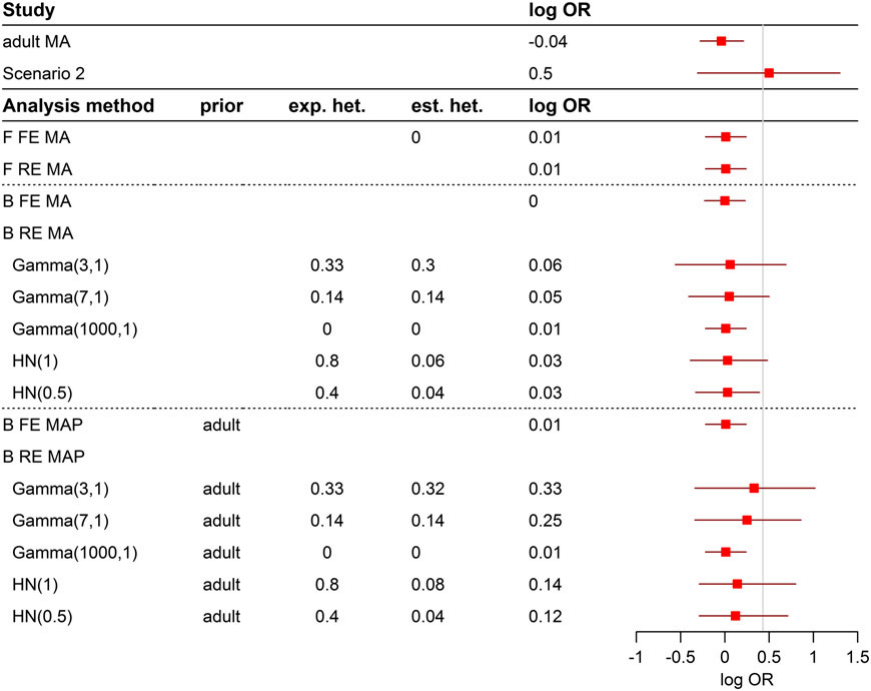
Results and conclusions for the comparison of immunosuppressive strategies in the heterogenious case

The analysis approaches introduced in Section 3 are applied to the examples in Section 2. Results are presented in Figures 1, 2, 3, with details on the effect estimate, corresponding 95% confidence or credible intervals and information on heterogeneity if available.

In Figure 1, results on the Bond and Opera examples are presented. The first 2 rows present the results of individual analyses of both studies followed by the frequentist (F) meta‐analysis (MA) results, both in the fixed‐effect (FE) and the random‐effects (RE) model together with the Q statistic and the estimated *τ*^2^. The estimated log ORs are quite similar in both models, and both models indicate that it may be unwise to combine the studies for calculation of a joint treatment effect: In the fixed‐effects model the overall treatment effect is significant, the conventionally significant heterogeneity statistics (*P* = .039), however, draws the underlying model assumptions into question. In contrast, in the random‐effects model due to the incorporation of the heterogeneity into the estimated variance, the treatment effect is no longer significant. Implications of this have been discussed in a previous paper.^31^

The Bayesian fixed‐effect approach leads to results that are virtually identical to the frequentist's fixed‐effect outcome due to the specified uninformative prior distributions. In the Bayesian random‐effects meta‐analysis model, it is possible to incorporate additional belief about the degree of heterogeneity using the priors on *τ*^2^ as introduced in Section 3.4. The example clearly demonstrates that the assumption of higher heterogeneity is directly reflected in wider 95% credible intervals. Posteriors of the heterogeneity parameter show that the model is not able to really change the a priori assumption of high or low heterogeneity if only 2 studies are included in the meta‐analysis.

The results of the Bayesian MAP approach are presented in the last rows. The significant first study, Bond, is incorporated as prior information in the analysis of the Opera study as explained in Section 3.3. The fixed‐effect model results are again almost identical to the frequentist fixed‐effect results. In contrast, the results of the random‐effects model move from close to the estimate in the new study (log OR = 0.18) towards the weighted fixed‐effect estimate of both studies (log OR = 0.46) depending on the prespecified degree of heterogeneity. Again, the prior belief in the degree of heterogeneity cannot be changed in the 2‐study situation. Here, with an equal amount of information in both studies, the MAP approach leads to an almost equal weighting of the individual study estimates in case no heterogeneity is assumed for the prior distribution of heterogeneity. If this is not the case, more weight is given to the new study, and only little information is borrowed from the historical information obtained from the Bond trial. It is noted that the assumption about heterogeneity determines the degree of borrowing of information and the conclusions and not the observed discrepancy of results in the old and the new studies. This introduces a subjective element into the overall results, which, in the regulatory context, requires at the minimum, an upfront justification (and a post hoc assessment of implications, if prior assumptions seem to not hold true).

Results for the example of paediatric extrapolation for immunosuppressive treatment are presented in Figures 2 and 3 for the homogenous and heterogeneous scenario, respectively. Again, results of individual studies to be included in the extrapolation exercise are displayed in the upper part of the figure. All studies were conducted as noninferiority studies, and the proposed noninferiority margin of 0.43 on the log OR scale (represented by a vertical line) guides assessment and interpretation of the results.

In the homogeneous scenario (Figure 2, adult and paediatric treatment are in full agreement), both the frequentist fixed‐effect and random‐effect MA lead to equal results and noninferiority of the new treatment strategy as compared with control. Trivially, the estimate for *τ*^2^ is zero.

The Bayesian fixed‐effect MA is again identical to the frequentist results, as there is no heterogeneity and a noninformative or vague prior distribution on the overall effect has been used. Bayesian random‐effects models with different informative prior assumptions on the heterogeneity parameter are used in the analysis. Although the experiment was conducted under the homogeneity assumption, an assumption of substantial heterogeneity in the analysis will lead to wide credible intervals that span the noninferiority margin and thus would not allow concluding efficacy/ability to extrapolate to the paediatric population. Again, the width of the credible intervals strongly depends on the assumption about the degree of heterogeneity, but the estimate for the treatment effect remains unchanged as compared with the other analyses in the frequentist and Bayesian meta‐analysis models.

The treatment effect estimate from the meta‐analysis of the adult studies is used as the prior information in the Bayesian MAP approaches. The fixed‐effect MAP approach shows again almost the same results as the fixed‐effect models. Since the studies in the adult MA are homogeneous and the paediatric study results have been chosen to be homogenous as well, we do not see a shift in the treatment effect (as compared with the Bond and Opera examples).

Results of the scenario with the apparently heterogeneous outcome of the paediatric trial are presented in Figure 3. As in the homogeneous scenario, the frequentist MA methods and the Bayesian fixed‐effect MA lead to almost the same effect estimate, and based on the corresponding 95% confidence and credible intervals, noninferiority can be concluded. The present heterogeneity between the adult studies and the paediatric study cannot be detected by the heterogeneity test in the frequentist fixed‐effect MA (Q = 1.98, *P* = .577). The results and conclusions of the Bayesian random‐effects MA are again dependent on the a priori assumed heterogeneity. The use of a prior representing a high heterogeneity results in wide 95% credible interval, which includes the noninferiority margin of 0.43 so that noninferiority cannot be concluded.

The Bayesian fixed‐effect MAP approach leads again to the same estimate and conclusion as the other fixed‐effects models. A quite interesting picture becomes apparent when the random‐effects MAP approach is applied: In case high heterogeneity is assumed through the IG prior, the effect estimate is close to the paediatric study effect, and the prior based on the adult data has less weight in the estimation. In this situation, noninferiority cannot be concluded. This is also the case if the heterogeneity assumption is slightly lowered. If high heterogeneity is incorporated through a half‐normal prior, similarly wide credible estimates are observed, but the posterior distribution is skewed, and the adult information again gets more weight in the effect estimation. If no heterogeneity is assumed, the effect, as in the other examples, is equal to the fixed‐effect weighted average, and noninferiority can be concluded. It is again quite clear from the presented results that the assumption on heterogeneity by defining the prior distribution cannot be changed if 2 or even only one study is considered in the Bayesian random‐effect analyses.

## 5 DISCUSSION

Legislation changed in 2007 to acknowledge substantial limitations in the evidence on how to appropriately treat paediatric patients with available medicines. The Paediatric Committee was established to define requirements for licensing new medicines for children in the light of existing needs and knowledge gaps.^32^

Thereafter, all applications for marketing authorization for new drugs and variations for new indications have to include a Paediatric Investigation Plan unless an explicit waiver has been granted. Since then, as well, all stakeholders are confronted with a limited interest to conduct trials in children despite the acknowledged lacking information and a reluctance that is going beyond the scientifically well‐founded argument that unnecessary experimentation should be avoided in general, and not only in a particularly vulnerable patient population.

The idea of incorporating historical (external) data into the assessment of a current clinical trial to reduce the burden of evidence required for decision‐making dates back to Pocock.^11^ He proposed that available information for the control arm should be used in a new trial to reduce the number of subjects under control and to have the opportunity to include more patients in the experimental treatment arm. Pocock^11^ specified conditions to ensure the comparability between the historical data and the current trial to reduce the risk of bias introduced into the evaluation of the trial outcome while incorporating historical data, essentially assuring that own data are used for decision‐making. However, using exclusively the information of the control arm will increase the risk of bias by means of increasing the dependency on the patient population of the historical trial. For this reason, the standard approach in meta‐analysis is to combine treatment effects (ie, the difference between treatment groups) from individual trials and not the outcome in each treatment group because it can be assumed that the treatment effect in a randomized trial is more stable as baseline characteristics should be balanced. The same considerations should apply in the extrapolation context, as well.

Extrapolation from an adult development programme to the paediatric setting is a very specific context for a number of reasons, but specifically that available data in adults have justified a licensing decision. Therefore, it can be assumed that the mechanism of action, the chosen dosing regimen, and other particularities of the treatment application (drug‐drug interactions etc) have been thoroughly investigated and are well understood. Based on investigation into the similarity of disease, drug pharmacology, and/or clinical response between adults and paediatrics, an agreement between stakeholders (usually the manufacturer of the drug and the drug licensing agencies) can be made that extrapolation from adults to paediatric patients is a plausible approach. Thereafter, the scientific questions to be addressed can be agreed and can be translated into study objectives and designs, including criteria to judge the success of the extrapolation exercise and the totality of evidence available. It can be sufficient to demonstrate consistency of outcome in the paediatric patient population with the well‐established information in adults, rather than generating a self‐standing formal proof of efficacy in paediatric patients. Alternatively, these considerations could be the basis for a statistical borrowing of external information for the assessment of the current clinical trial. In these cases, a scientific rationale exists, beyond lack of feasibility for recruitment to trials, to use existing data in drawing inferences about effects in paediatric patients.

The availability of data with the medicinal product under investigation for the treatment of the same disease is a strength of the extrapolation situation and sharply contrasts with the situation of rare disease, where usually at best information on a similar product in a similar context is available and prior knowledge expresses to a greater extent credibility than evidence. This may justify the application of methodology that can incorporate this credibility into the assessment,^33^ but it is appropriate to acknowledge that limits for the generation of direct evidence exist and, in consequence, decision‐making has to be done with higher uncertainties.

Bayesian MAP methods have been proposed as one approach to incorporate prior knowledge into the assessment of new data and therefore more or less correct the observed paediatric data under prior assumptions that can be based on already available adult data. The prior is an implicit means of reducing the amount of prospective data to be generated for a formal decision‐making process.

Based on principles of drug regulation, preference is given to data‐based decision‐making and, in consequence, to data‐based priors in the Bayesian context. Both, a classical meta‐analysis incorporating the adult data and the final paediatric data to arrive at a summary frequentist (or Bayesian) estimate for the treatment effect, and a MAP approach are methods to arrive at an overall assessment of new data in combination with previously generated data.

This comparison is enlightening because current practice in (sequential) meta‐analysis would always assess previous knowledge (the prior in the Bayesian context), the newly generated information (the current paediatric trial), and the combined information (the outcome of the meta‐analysis), separately. Discussion of the consistency of these pieces of information is an increasingly important aspect of the assessment of the credibility of findings and conclusions in the field of meta‐analysis. It is an important principle in the regulatory assessment of clinical trials to assess each piece of information separately before combining the independent pieces for decision‐making. This is in contrast to the Bayesian strategy that emphasizes the justification of the prior and the evaluation of the overall outcome, but not the newly generated information as an independent piece of information.

Obviously then 2 aspects have to be determined: the overall amount of evidence required and the degree of consistency needed to reassure that successful extrapolation to the paediatric population is plausible. The comparison with the meta‐analytical approach also supports to discuss aspects of weighting as it is well known that whenever information is combined with weighted estimates, there is a risk of trivial conclusions in those situations where different amounts of information are combined. In all instances of paediatric extrapolation, a large body of evidence is available in the adult setting and would eventually be combined with limited paediatric information, whenever clinical extrapolation is attempted. The ability to easily calculate weights and contributions to a heterogeneity statistics makes the frequentist fixed‐effects meta‐analysis model a means to discuss this aspect of combination of independent pieces of information. We have investigated 2 examples with an unequal amount of information between the populations. In one scenario, the paediatric data were completely in line with the adult data, which represents the alternative hypothesis of the extrapolation exercise under the assumption that disagreement between the adult and the paediatric data is effectively the null hypothesis of the decision problem. In the second scenario, we have investigated the situation that this null hypothesis is true. Irrespective of the scenario, when using a traditional meta‐analysis approach for arriving at a conclusion regarding efficacy, the amount of adult data by far exceeds the available and usually limited paediatric data, and in consequence, the summary estimates will always be dominated by the adult data.

We have shown that the question whether (or not) a positive conclusion on the ability to extrapolate to paediatric data depends on the assumption about heterogeneity of the treatment effects. An a priori assumption of substantial heterogeneity leads to even wider credible intervals than the confidence intervals of the frequentist's random‐effects meta‐analysis. If no heterogeneity is assumed, the results are identical to a replication of the fixed‐effect meta‐analysis outcome, and in both instances, the conclusion in the Bayesian context is independent of the true heterogeneity. This can also be seen in the work of Friede et al,^34^ who recommend based on simulations a Bayesian meta‐analysis in the case of 2 studies over frequentist approaches.

This does, however, not address the issue that in situations with studies having different weights, the assumption about heterogeneity and not the observed/true discrepancy or agreement of studies will in most instances determine the outcome of the assessment of the summary estimate. In addition, neither homogeneity nor heterogeneity of studies can be detected by an assessment of the overall estimate.

In consequence, based on gaps in knowledge and uncertainties on the relevance of the data from adults, thorough calculation is needed at the planning stage for how much data in paediatrics need to be generated so that the final assessment can detect or exclude relevant differences between the adult and the paediatric data. This discussion is independent from the chosen methodology.

Therefore, it is of huge importance that the derivation of the prior distributions and their impact on the analysis results are well understood. Until now, there is no real consensus about a possible approach in this matter. Turner et al^35^ propose the elicitation of prior distributions for heterogeneity based on previous meta‐analyses in the same therapeutic field. Similarly in the example of immunosuppressive treatment heterogeneity of the adult studies could be used to inform about the general heterogeneity of the therapeutic situation. However, this would miss the point that irrespective of whether the adult studies are homogeneous or not, the heterogeneity to the outcome of the paediatric trial is the interest of the extrapolation exercise. An application of this approach in the immunosuppressive treatment example would lead to a prior that does not allow for heterogeneity, and a fixed‐effect meta‐analysis would be pre‐planned for the analysis. However, at the planning stage, the degree of heterogeneity as well as the paediatric data is still unknown, and thus, in the current example, trivial conclusions would be unavoidable with the currently planned sample size of the paediatric trial.

The observation that there are in general more difficulties with scale parameters than location parameters in Bayesian analyses has also been stated by Lambert et al.^30^ They also state that this changes with an increasing amount of actually available evidence, which is a solution of only limited relevance for paediatric extrapolation due to the fact that the similarity of the paediatric study outcome is actually the objective of the paediatric programme and the reluctance to conduct larger paediatric studies.

Downweighting of the adult information has been proposed by a number of authors (eg, previous studies^1^, ^36^) and essentially is accomplished by assuming substantial heterogeneity in the Bayesian analyses. This introduces an arbitrary element and is to at least some extent contradictory in those situations where the historical study is a well‐planned, randomized, controlled, and blinded trial (thus presenting evidence of high internal validity). An alternative approach might be to argue for a content‐wise reduction of the adult information (eg, by simply taking the information from young adults for extrapolation to the adolescent population instead of using the full trial information). Other arguments to use a subpopulation of the adult studies for the extrapolation exercise may be found from scientific grounds (ie, selecting those trials that best fit regarding aspects of design, co‐medication, or in general intrinsic or extrinsic factors.

In our examples, we demonstrated that substantially different effect estimates, respective confidence and credible intervals, and conclusions are possible if Bayesian analyses are applied. Prespecification and prespecified decision‐making are important aspects in drug regulation, and it is therefore of paramount importance to precisely prespecify the assumptions that are incorporated explicitly and implicitly in such analyses. Bayesian methodology can be one tool in the statistician's armoury for scenarios where there is agreement that based on the relevance of available data in a source population, a self‐standing formal proof of efficacy is not needed. Nonetheless, efficacy data generated in the target population should be given appropriate weight in analysis and inference and trivial conclusions should be avoided through careful planning of the exercise.

## FUNDING

This work has been partly funded by the FP7‐HEALTH‐2013‐INNOVATION‐1 project: Advances in Small Trials Design for Regulatory Innovation and Excellence (ASTERIX), grant agreement no. 603160.
